# *BRCA1/2* and *TP53* mutation status associates with *PD-1* and *PD-L1* expression in ovarian cancer

**DOI:** 10.18632/oncotarget.24770

**Published:** 2018-04-03

**Authors:** Verena Wieser, Inge Gaugg, Martina Fleischer, Giridhar Shivalingaiah, Soeren Wenzel, Susanne Sprung, Sigurd F. Lax, Alain G. Zeimet, Heidelinde Fiegl, Christian Marth

**Affiliations:** ^1^ Department of Obstetrics and Gynecology, Medical University of Innsbruck, Innsbruck 6020, Austria; ^2^ Division of Human Genetics, Medical University of Innsbruck, Innsbruck 6020, Austria; ^3^ Institute of Pathology, Medical University of Innsbruck, Innsbruck 6020, Austria; ^4^ Department of Pathology, Hospital Graz Süd-West, Academic Teaching Hospital of the Medical University Graz, Graz 8020, Austria; ^5^ Present address: Division Biological Chemistry, Biocenter, Innsbruck, Medical University of Innsbruck, Innsbruck 6020, Austria

**Keywords:** ovarian cancer, PD-1, PD-L1, IFNγ, BRCA1/2

## Abstract

Checkpoint molecules such as programmed cell death protein-1 (PD-1) and its ligand PD-L1 are critically required for tumor immune escape. The objective of this study was to investigate tumoral *PD-1* and *PD-L1* mRNA-expression in a cohort of ovarian cancer (OC) patients in relation to tumor mutations. We analyzed mRNA expression of *PD-1*, *PD-L1* and *IFNG* by quantitative real-time PCR in tissue of 170 patients with low grade-serous (LGSOC), high-grade serous (HGSOC), endometrioid and clear cell OC compared to 28 non-diseased tissues (ovaries and fallopian tubes) in relation to tumor protein 53 (*TP53*) and breast cancer gene 1/2 (*BRCA1/2*) mutation status. *TP53*-mutated OC strongly expressed *PD-L1* compared to *TP53* wild-type OC (*p* = 0.028) and *BRCA1/2*-mutated OC increasingly expressed *PD-1* (*p* = 0.024) and *PD-L1* (*p* = 0.012) compared to *BRCA1/2* wild-type OC. For the first time in human, we noted a strong correlation between tumoral *IFNG* and *PD-1* or *PD-L1* mRNA-expression, respectively (*p* < 0.001). OC tissue increasingly expressed *PD-1* compared to healthy controls (vs. ovaries: *p* < 0.001; vs. tubes: *p* = 0.018). *PD-1* and *PD-L1* mRNA-expression increased with higher tumor grade (*p* = 0.008 and *p* = 0.027, respectively) and younger age (< median age, *p* = 0.001). Finally, in the major subgroup of our cohort, FIGO stage III/IV HGSOC, high *PD-1* and *PD-L1* mRNA-expression was associated with reduced progression-free (*p* = 0.024) and overall survival (*p* = 0.049) but only in the univariate analysis. Our study suggests that in OC *PD-1*/*PD-L1* mRNA-expression is controlled by *IFNγ* and affected by *TP53* and *BRCA1/2* mutations. We suggest that these mutations might serve as potential predictive factors that guide anti-*PD1*/*PD-L1* immunotherapy.

## INTRODUCTION

Ovarian cancer (OC) is the major cause of death among gynecological cancer entities [1]. In recent years, multidisciplinary treatment options including surgery, chemotherapy regimens and anti-angiogenic agents have considerably evolved [2], however, long term prognosis for OC patients remains devastating [3]. Therefore, therapies targeting tumor immunogenicity and anti-tumor immunity [4] such as antibodies that inhibit checkpoint molecules, i.e. the programmed cell death ligand-1 (PD-L1)/programmed cell death-1 (PD-1) pathway [5] have recently gained attention as a novel therapeutic option in OC [6].

PD-L1 is expressed by tumor cells [7] to inactivate T-cells via binding to PD-1 [8] and escape from the immune system [7]. Checkpoint (i.e. PD-1/PD-L1) inhibitors can restore T-cell mediated tumor immunogenicity and have been successfully established in anti-tumor treatment [9]. Ongoing clinical trials investigate whether PD-1/PD-L1 inhibitors can be an effective treatment option for patients with OC.

The rationale to test the efficacy of checkpoint inhibitors in OC arises from the observation that intratumoral T-cells directly correlate with clinical outcome [10] and that the PD-1/PD-L1 pathway may play a relevant role in the immune evasion of malignant ovarian tumors [11]. Clinical trials with immune checkpoint inhibitors were performed in patients with advanced and recurrent OC and demonstrated response rates of ∼15% [6]. Thus, checkpoint inhibitors may improve clinical outcome in a subgroup of OC patients, but predictors of response or identification of patients who benefit are urgently needed.

In the present study we, therefore, systematically analyzed *PD-1* and *PD-L1* mRNA expression in 170 epithelial OC in relation to 28 non-neoplastic tissues and to clinicopathological features to identify a subgroup of patients which may profit by checkpoint inhibitors. Since *IFNγ* was found to play an essential role in the adaptive immune resistance of tumors as an inducer of *PD-L1* on tumor cells [12], i.e. on ovarian cancer cells *in vitro* [13], we further performed correlation analyses between *IFNγ* and *PD-1* or *PD-L1* to investigate the regulative role of the PD-1 pathway in OC. Previous studies demonstrate that tumors with high mutational burdens exhibit a greater response rate to immune checkpoint blockade [14–16]. Based on these observations we further stratified our analysis by *BRCA1/2* and *TP53* mutation status.

## RESULTS

### *PD-1* expression is elevated in OC tissue and fallopian tubes

**Figure 1:**
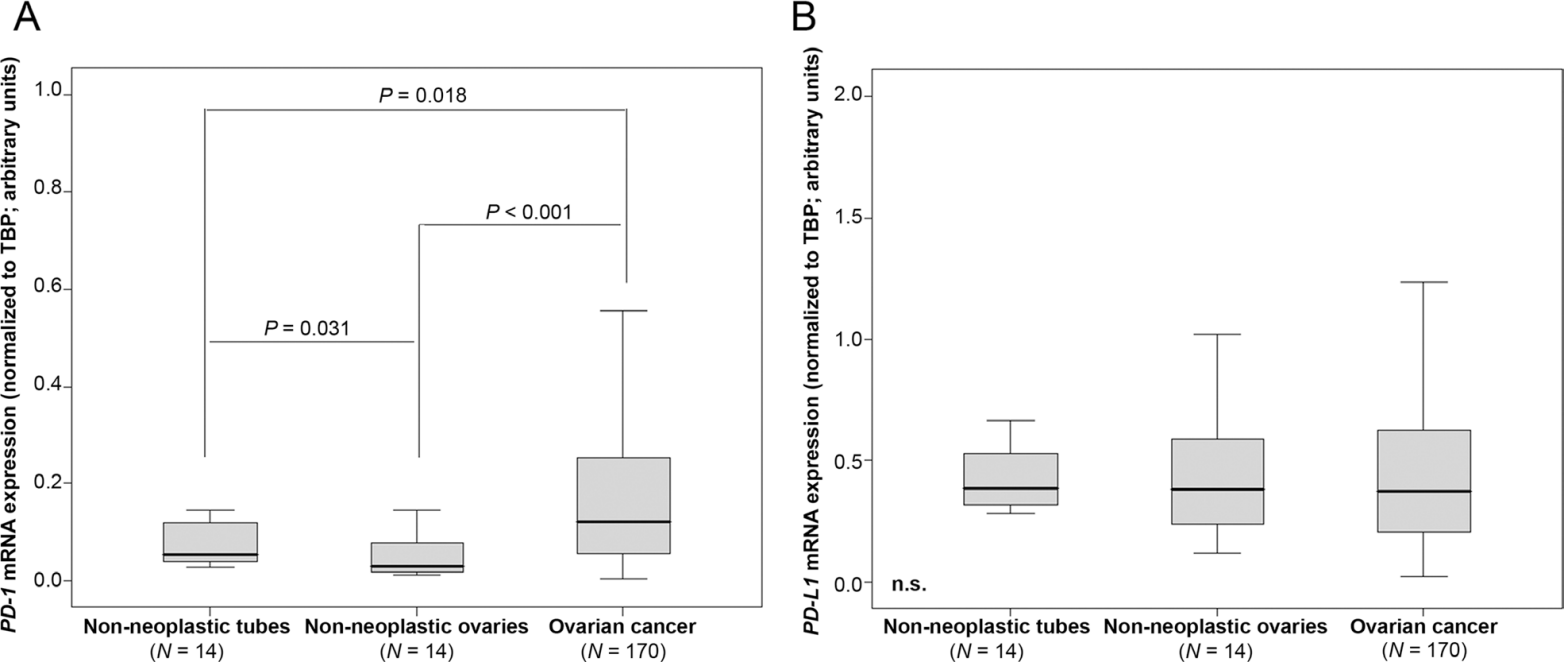
*PD-1* mRNA expression is elevated in OC tissue compared to non-neoplastic ovaries and fallopian tubes (**A**) *PD-1* expression in non-neoplastic fallopian tubes, non-neoplastic ovaries and OC. (**B**) *PD-L1* expression in non-neoplastic fallopian tubes, non-neoplastic ovaries and OC. *PD-1* and *PD-L1* mRNA expression values were normalized to *TBP* expression.

To evaluate the potential regulative power of the PD-1 pathway in OC, we analyzed mRNA expression levels in cancer tissue and non-neoplastic ovaries and fallopian tubes. We determined strong *PD-1* expression in cancer tissue compared to non-cancer tissues (OC vs non-neoplastic ovaries: *p* < 0.001; OC vs. non-neoplastic tubes: *p* = 0.018; Figure 1A). We further found higher expression of *PD-1* in non-neoplastic tubes compared to non-neoplastic ovaries (*p* = 0.031; Figure 1A). However, we did not note increased *PD-L1* expression in OC tissue compared to non-neoplastic tissues (Figure 1B). Detection of PD-L1 by immunohistochemistry was associated with increased *PD-L1* expression determined by qPCR in non-malignant tissues (Supplementary Figure 1).

### *PD-1* and *PD-L1* mRNA expression strongly correlates with *IFNG* mRNA expression

**Figure 2:**
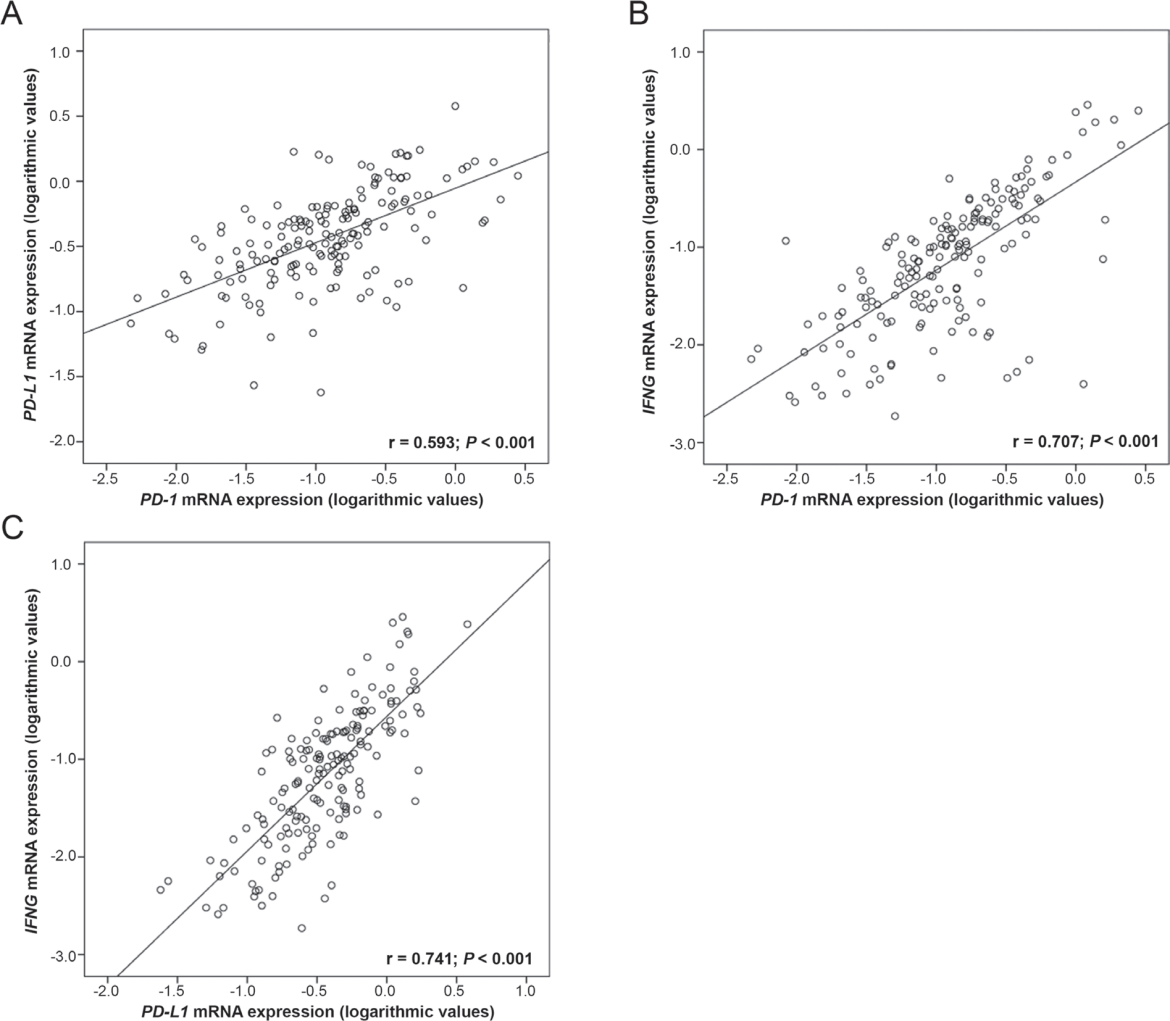
Correlation of *PD-1* and *PD-L1* mRNA expression with *IFNG* mRNA expression in 170 OC tissues Linear regression analysis of (**A**) *PD-1* and *PD-L1*, (**B**) *PD-1* and *IFNG* and (**C**) *PD-L1* and *IFNG*. *PD-1*, *PD-L1* and *IFNG* mRNA expression values were normalized to *TBP* expression.

Performing Spearman rank association analyses of 170 OC tissues, we noted a significant correlation of *PD-1* with *PD-L1* expression (*p* < 0.001; r_S_ = 0.593). We further found a strong correlation between *IFNG* mRNA expression with both *PD-1* (*p* < 0.001; r_S_ = 0.707) and *PD-L1* (*p* < 0.001; r_S_ = 0.741). This was similarly demonstrable in our log-transformed data set with Pearson correlation analyses (Figure 2). These correlations were also detected in control tissues (data not shown).

### Increased *PD-1* and *PD-L1* mRNA expression occurred in young patients and high tumor grade

Next, we analyzed *PD-1* and *PD-L1* mRNA expression according to clinical and histopathological characteristics. We noted increasing *PD-L1* expression in younger (< median age of 60.4 years) patients (*p* = 0.001; Table 1A). Furthermore, *PD1* and *PD-L1* expression progressed with tumor grade (*p* = 0.008 and *p* = 0.027, respectively; Table 1A).

**Table 1:** Association of *PD-1* and *PD-L1* mRNA expression with clinicopathological features in ovarian cancer patients. Analysis in (**A**) all 170 ovarian cancer patients, (**B**) only HGSOC patients (*N* =106) and (**C**) only HGSOC, FIGO III/IV patients (*N* = 85)

(A) All tumors								
Variable		*n*	PD-1 mRNA expression (rel. to TBP)			PD-L1 mRNA expression (rel. to TBP)		
			Median	IQR	*P* value	Median	IQR	*P* value
**Age (median)**	≤60.4 yrs.	85	0.12	0.07–0.34	0.128	0.47	0.27–0.76	**0.001**
	>60.4 yrs.	85	0.13	0.05–0.21		0.32	0.16–0.53	
**FIGO stage**	I	38	0.11	0.05–0.32	0.350	0.38	0.15–0.68	0.563
	II	13	0.10	0.05–0.51		0.33	0.20–1.01	
	III	102	0.12	0.06–0.21		0.37	0.22–0.58	
	IV	17	0.23	0.13–0.39		0.51	0.25–0.95	
**Tumor grade**	1	12	0.10	0.06–0.17	**0.008**	0.38	0.24–0.47	**0.027**
	2	81	0.10	0.04–0.20		0.32	0.17–0.51	
	3	77	0.16	0.08–0.37		0.47	0.23–0.78	
**Residual disease**	no	78	0.11	0.04–0.23	0.113	0.35	0.17–0.58	0.090
**after surgery**	yes	87	0.14	0.08–0.31		0.45	0.24–0.70	
	unknown	5						
**Histology**	HGSOC	106	0.12	0.05–0.22	0.219	0.33	0.20–0.59	0.131
	LGSOC	11	0.09	0.06–0.15		0.38	0.23–0.42	
	endometroid	43	0.14	0.05–0.38		0.49	0.20–0.68	
	clear cell	10	0.28	0.07–0.42		0.60	0.36–1.07	

(B) HGSOC								
Variable		*n*	PD-1 mRNA expression (rel. to TBP)			PD-L1 mRNA expression (rel. to TBP)		
			Median	IQR	*P* value	Median	IQR	*P* value
**Age (median)**	≤60.9 yrs.	53	0.11	0.05–0.27	0.451	0.46	0.24–0.75	**0.004**
	>60.9 yrs.	53	0.13	0.04–0.19		0.28	0.15–0.44	
**FIGO stage**	I	14	0.12	0.05–0.29	0.213	0.24	0.11–0.91	0.157
	II	7	0.07	0.05–0.37		0.33	0.19–1.24	
	III	71	0.10	0.05–0.19		0.32	0.20–0.51	
	IV	14	0.21	0.14–0.30		0.54	0.34–0.94	
**Residual disease**	no	37	0.10	0.04–0.20	0.200	0.27	0.14–0.54	0.062
**after surgery**	yes	64	0.13	0.07–0.26		0.39	0.24–0.63	
	unknown	5						

(C) Only HGSOC, FIGO III/IV								
Variable		*n*	PD-1 mRNA expression (rel. to TBP)			PD-L1 mRNA expression (rel. to TBP)		
			Median	IQR	*P* value	Median	IQR	*P* value
**Age (median)**	≤59.2 yrs.	43	0.12	0.06–0.24	0.499	0.47	0.24–0.70	**0.021**
	>59.2 yrs.	42	0.13	0.04–0.19		0.31	0.20–0.45	
**FIGO stage**	III	71	0.10	0.05–0.19	**0.031**	0.32	0.20–0.51	**0.031**
	IV	14	0.21	0.14–0.30		0.54	0.34–0.94	
**Residual disease**	no	19	0.05	0.03–0.19	0.063	0.27	0.16–0.51	0.100
**after surgery**	yes	61	0.13	0.08–0.25		0.40	0.24–0.63	
	unknown	5						

Note: The significance level (P) was determined by Mann–Whitney U or Kruskal–Wallis test respectively.

Abbreviations: HGSOC, high-grade serous ovarian cancer; IQR, Interquartile range; LGSOC, low-grade serous ovarian cancer.

### *PD-1* and *PD-L1* mRNA expression is elevated in FIGO IV OC

When analyzing the subgroup of patients suffering from HGSOC (Table 1B) we further observed a tendency of higher *PD-L1* levels in patients with residual disease after primary debulking operation (Table 1B) as compared to patients with no macroscopic disease after upfront debulking. In advanced stage HGSOC higher *PD-1* and *PD-L1* mRNA expression was observed in tissues of stage IV when compared to stage III (*p* = 0.031, Table 1C).

### *BRCA1*/*2* and TP53 mutated tumors are associated with high *PD-1* and *PD-L1* levels

**Figure 3:**
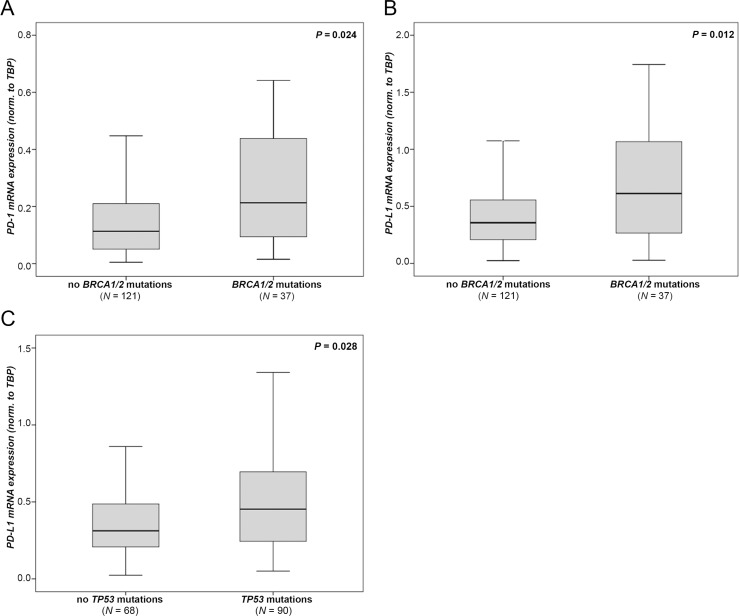
*PD-1* and *PD-L1* mRNA expression according to genetic aberrations *BRCA1/2* mutation data in association with (**A**) *PD-1* expression and (**B**) with *PD-L1* expression. (**C**) *TP53* mutation data in association with *PD-L1* expression. Expression values were normalized to *TBP* expression.

In 158 patients from our cohort, mutation analysis data for *BRCA1, BRCA2 and TP53,* genes known to account for OC, were available. We analyzed these cases for the association between gene mutations and *PD-1* and *PD-L1* expression. In 37 of these 158 (23.4%) OC cases, *BRCA1* or *BRCA2* (*BRCA1/2*) mutations were detected. In *BRCA1*/*2* mutated tumors, we found significant higher levels of *PD-1* (*p* = 0.024; Figure 3A) and *PD-L1* (*p* = 0.012; Figure 3B) compared to *BRCA1/2* wild-type tumors. We were unable to detect differences between *BRCA1* aberrations such as c.4183C>T and c.1687C>T which were mostly enriched in our cohort (data not shown). In 91 of 158 OC cases (57.2%) *TP53* mutations were detected. These tumors exhibited higher *PD-L1* levels compared to tumors with wild type *TP53* (*p* = 0.028; Figure 3C). A subgroup analysis revealed that these effects in mutated OC were only observed in HGSOC (Supplementary Figure 2), but not in non-HGSOC cases (data not shown).

### High *PD-1* mRNA expression is associated with a poor prognosis

**Figure 4:**
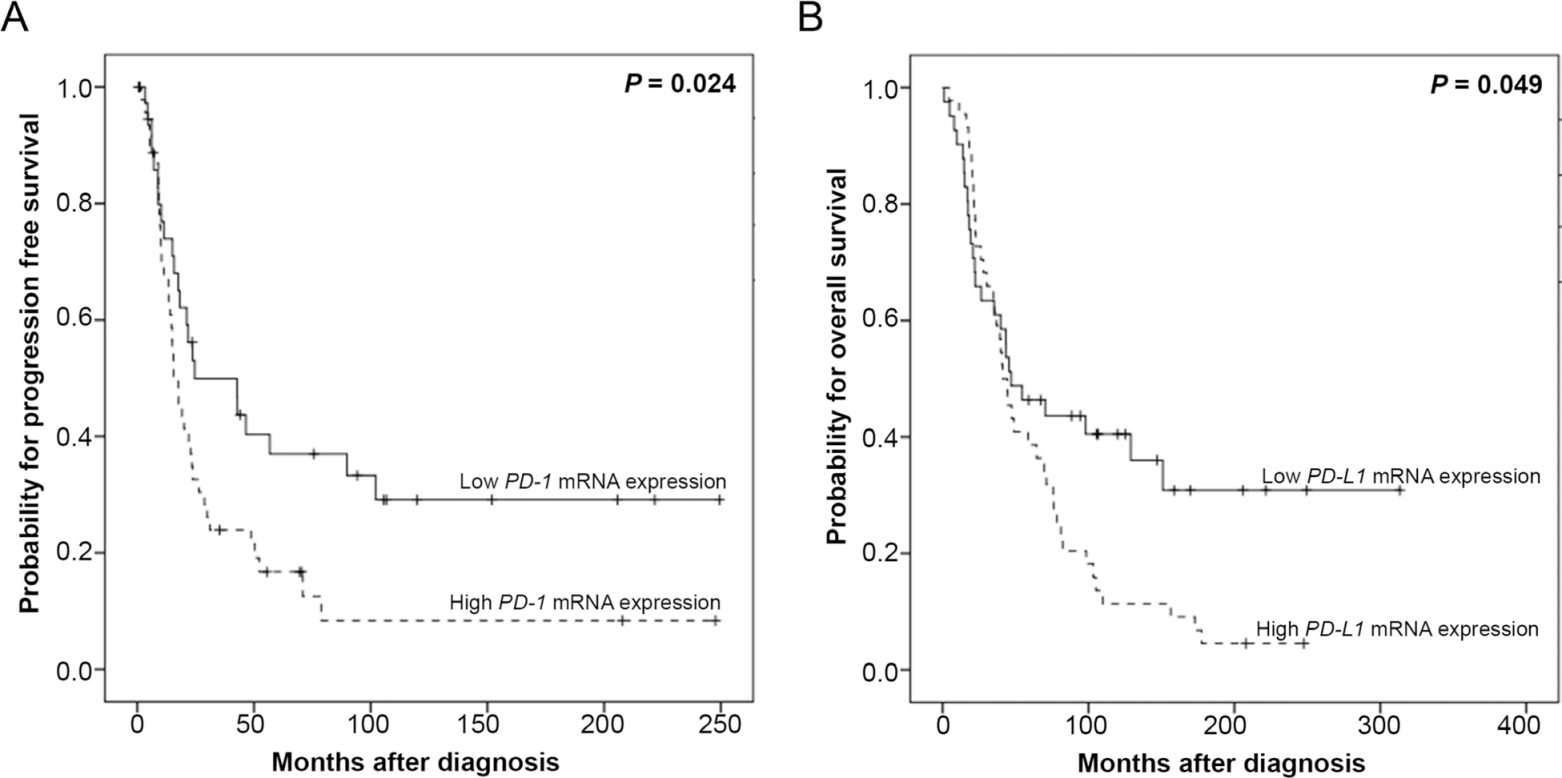
Kaplan–Meier survival analyses and *PD-1* and *PD-L1* mRNA-expression in 85 HGSOC, FIGO III/IV patients (**A**) *PD-1* mRNA expression and progression free survival. (**B**) *PD-L1* mRNA expression and overall survival.

We tested whether intratumoral *PD-1* or *PD-L1* expression was associated with clinical outcome. Therefore, we identified the optimal threshold for “high” and “low” expression using Youden’s index [17]. In the entire cohort, we could not observe a prognostic relevance of “*PD-1* high” or “*PD-1* low” expressing tumors (Table 2). However, when we analyzed patients with FIGO III/IV HGSOC, “*PD-1* high” expressing tumors were associated with significantly worse PFS (*p* = 0.024; Figure 4A, Table 2) when compared to “*PD-1* low” expressing tumors. More specifically, the median time to progression was 15.6 and 24.6 months for patients with “high” and “low” *PD-1* expression, respectively. Patients with “*PD-L1* high” expressing tumors exhibited a significant worse OS compared to patients with “*PD-L1* low” expressing tumors (*p* = 0.049; Figure 4B, Table 2). The median OS was 41.1 and 47.1 months for patients with “*PD-1* high” and “*PD-1* low” expressing tumors, respectively. However, the prognostic relevance of *PD-1* or *PD-L1* could not be confirmed in the multivariate Cox regression analyses (data not shown).

**Table 2:** Univariate survival analysis in 170 ovarian cancer patients. The optimal cutoff points for *PD-1* and *PD-L1* were calculated by the Youden’s index

Variable		Progression free Survival			Overall Survival		
		No. Patients (relapsed/ total)	Median, months (95% CI)	*P* value	No. Patients (died/total)	Median, months (95% CI)	*P* value
**Age (median)**	≤60.4 yrs.	51/85	45.5 (19.9–71.2)	0.685	48/85	107.2 (94.6–119.7)	**0.005**
	>60.4 yrs.	45/85	22.1 (8.2–35.9)		61/85	43.5 (33.4–53.6)	
**FIGO stage**	I/II	11/51	n.r.	**<0.001**	20/51	n.r.	**<0.001**
	III/IV	85/119	20.0 (14.7–25.3)		89/119	47.3 (26.6–68.0)	
**Tumor grade**	1/2	47/93	48.8 (0.0–101.6)	0.110	53/93	100.0 (70.1–129.9)	**0.012**
	3	49/77	23.6 (12.6–34.7)		56/77	44.4 (30.4–58.5)	
**Residual disease after surgery**	no	24/78	n.r.	**<0.001**	30/78	n.r.	**<0.001**
	yes	68/87	15.7 (13.2–18.3)		76/87	35.2 (24.4–46.1)	
**Histology**	HGSOC	69/106	23.4 (17.4–29.4)	**0.008**	80/106	47.1 (27.5–66.7)	**0.003**
	others	27/64	n.r.		29/64	132.7 (n.r.)	
**PD-1 mRNA expression**	low	63/118	35.3 (14.2–56.5)	0.597	30/50	68.8 (0.0–140.8)	0.633
	high	33/52	23.4 (12.1–34.7)		79/120	71.1 (43.6–98.6)	
**Subgroup:**	low	40/69	24.6 (2.5–46.7)	0.286	24/36	45.6 (0.0–100.2)	0.212
**HGSOC**	high	29/37	20.0 (13.0–27.0)		56/70	47.1 (33.0–61.2)	
**Subgroup:**	low	23/38	24.6 (0.0–53.1)	**0.024**	16/25	45.6 (0.0–129.2)	0.088
**HGSOC, FIGO III/IV**	high	40/47	15.6 (11.0–20.2)		52/60	44.4 (34.9–53.9)	
**PD-L1 mRNA expression**	low	44/88	32.1 (6.2–58.0)	0.872	52/88	68.8 (20.0–117.7)	0.773
	high	52/82	30.0 (6.2–53.7)		57/82	74.9 (42.0–107.9)	
**Subgroup:**	low	28/55	22.8 (0.0–51.4)	0.202	33/54	49.6 (18.2–81.1)	0.094
**HGSOC**	high	41/51	23.4 (18.0–28.9)		47/52	44.4 (33.6–55.2)	
**Subgroup:**	low	24/42	18.2 (7.8–28.6)	0.159	26/41	47.1 (14.0–80.2)	**0.049**
**HGSOC, FIGO III/IV**	high	39/43	21.8 (14.4–29.2)		42/44	41.1 (32.6–49.7)	

Note: The significance level (P) was determined by log-rank test.

Abbreviations: n.r., not reached.

## DISCUSSION

In this study, we investigated the expression of checkpoint molecules in a Caucasian OC cohort. We found that *TP53* and *BRCA1/2* mutated OC was associated with high *PD-1* and *PD-L1* expression. Both, *PD-1* and *PD-L1* expression correlated with *IFNG* which is known to induce checkpoint molecules in OC cells [13]. We also noted that young women and patients with advanced OC exhibited increased expression of *PD-1* and *PD-L1* which was associated with poor clinical outcome.

Mutations in *BRCA1/2* and *TP53* confer a significant lifetime risk for OC and are considered a major driver of tumorigenesis [18]. Tumors that exhibit these mutations usually present a high neoantigen load [19]. In our cohort, we demonstrate that *BRCA1*/*2* mutated tumors exhibit high *PD-1* and *PD-L1* levels supporting the notion that *BRCA1/2*-mutated tumors may be more sensitive to PD-1/PD-L1 inhibitors compared to wild-type tumors [19] and that the combination therapy of checkpoint inhibitors with poly (ADP-ribose) polymerase (PARP) inhibitors may be more successful [19]. Consistent with previous data we found *TP53* mutations in 66% of HGSOC which is within the range of 50–80% as previously reported [20]. In line with previous studies on lung adenocarcinoma [21] we found that *TP53* mutated tumors showed higher *PD-L1* expression compared to *TP53* wild-type tumors. Dong *et al.* suggested that *TP53* mutated lung adenocarcinomas with increased mutation burden showed remarkable clinical benefit to PD-1 inhibitors [21]. In line with these results, we suggest that the *TP53* mutation status can be used as a potential surrogate predicting treatment response in patients receiving anti-PD-1/PD-L1 immunotherapy.

Checkpoint molecule expression is known to be a negative predictor for clinical outcome in various cancer entities [22]. The available data on the prognostic significance of *PD-1* and *PD-L1* expression in OC are inconsistent. We found that patients with FIGO III/IV HGSOC and “*PD-L1* high” expressing tumors exhibited worse OS when compared to patients with “*PD-L1* low” expressing tumors. Our findings are in line with the recent report by Hamanishi *et al.* who demonstrated that the engagement of the PD-1 pathway (i.e. *PD-L1* expression) is associated with a poor prognosis in OC patients [11]. In contrast, Darb-Esfahani *et al.* demonstrated a favorable prognosis for HGSOC patients with immunohistochemically or transcriptionally high PD-1 and PD-L1 expression in lymphocytes and cancer cells, respectively [23]. Another study by Webb *et al.* also demonstrated that immunohistochemical PD-L1 labeling (primarily of macrophages) correlated with lymphocytic infiltration and improved survival in patients with HGSOC [24]. In other tumor entities such as lung cancer, colorectal cancer and melanoma, *PD-L1* expression was shown to have both positive and negative prediction value which may be due to the following reasons [22]: Primer-based detection of *PD-1* and *PD-L1* has technical issues, such as variable primer sequences, tissue preparation, processing variability or different statistical cut-offs resulting in misleading expression status. Furthermore, *PD-1* and *PD-L1* expression in tumors is affected by temporal (i.e. infections, co-medication such as corticosteroids) and spatial factors, leading to erroneous interpretation of the results. However, our data indicate that high *PD-1* or *PD-L1* expression are negative prognosticators in OC in line with observations of various other tumor entities [25–27].

Mechanistically, PD-L1 expression was shown to be induced by IFNγ stimulation in mammalian OC cells thereby triggering a negative feedback on T cell activity [13]. This study extensively investigated IFNγ-dependent upregulation of PD-L1 on OC cells. They examined PD-L1 expression on several human and mouse ovarian cancer cell lines by flow cytometry: First, the SK-OV-3 and OVCA429 human OC cell lines already express high levels of PD-L1 at baseline, while the OVARY1847 human OC cell line strongly expressed PD-L1 after exposure to human recombinant IFNγ. Furthermore, the injection of IFNγ into tumours induced PD-L1 expression and promoted tumour growth, while PD-L1 inhibition abrogated tumour growth. In agreement with these findings, we found a strong positive correlation between *IFNG* and *PD-L1* and also *PD-1* expression in human OC. These data support the idea that IFNγ released by the tumor microenvironment may be involved in tumor immune escape [28], possibly via the upregulation of checkpoint molecules [13]. Given that we further found a positive correlation between *IFNG*, *PD-L1* and *PD-1* in non-malignant tissue (data not shown) it appeared that IFNγ regulated PD-1 and PD-L1 expression independent of benign, inflamed or malignant conditions as demonstrated previously [29, 30]. However, we suggest that checkpoint molecule expression and tumor escape mechanisms are highly relevant in the latter condition as demonstrated by above mentioned studies [7, 12].

As our findings were based on mRNA expression determined by qPCR, we aimed at verifying that transcription correlated with translation of checkpoint molecules in our cohort. Histologically ∼80% of the analyzed OC tissue were composed of cancer cells which led us to conclude that *PD-L1* expression mostly originated from malignant OC cells. While we observed an association between *PD-L1* mRNA and protein expression in non-malignant tissues, we did not observe such association in OC (data not shown). This may be explained by intra-tumor heterogeneity and the technical issue of sample preparation for qPCR and immunohistochemistry analysis from two different sites of the tumor in our study. As a direct relationship between *PD-L1* mRNA and protein expression was not demonstrable in OC, and immunohistochemistry only allows protein quantification to a limited extent, we analyzed clinical characteristics with the results from qPCR expression. However, based on the association between *PD-L1* RNA expression and protein level from non-malignant tissue, we believe that qPCR is a reliable tool for our analyses [31]. We were unable to determine PD-1 protein expression by immunohistochemistry due to lack of available antibodies that yielded a specific signal in our laboratory.

While we demonstrated that *PD-1* expression is significantly higher in OC compared to non-neoplastic ovaries and Fallopian tubes, we did not observe increased *PD-L1* mRNA expression in cancer tissue compared to non-diseased ovaries or tubes. Increased *PD-1* expression may be explained by a study from Webb *et al.* which demonstrated limited PD-1 protein expression on infiltrating lymphocytes in healthy fallopian tubes, but strong PD-1 expression in tumor infiltrating lymphocytes in OC tissue [32]. In contrast, PD-L1 is a cell surface protein that is not only expressed by tumor cells but also by activated antigen-presenting cells which may affect *PD-L1* expression of non-malignant tissues [30]. For example, Maine *et al.* demonstrated that *PD-L1* is strongly expressed on ascites-derived monocytes [33]. We acknowledge that the authors in this study observed increased PD-L1 expression in OC compared to healthy controls which is not demonstrable in our cohort.

In summary, *PD-1* and *PD-L1* expression emerged as critical determinant of OC progression especially in young patients (i.e. < median age of 60.4 years) with *BRCA1/2* or *TP53* mutated OC. These findings suggest an involvement of checkpoint regulation in OC progression. Our data may guide OC treatment by check point inhibition in the future [34].

## MATERIALS AND METHODS

### Patients and samples

Ovarian tissue samples from 170 patients with OC obtained at primary debulking (patients were 24 to 90 years old; median age at diagnosis was 60 years) and control tissues from 28 patients obtained by elective salpingo-oophorectomy for benign conditions (14 non-neoplastic tubal tissues: patients were 30 to 73 years old, median age: 50 years; 14 non-neoplastic ovaries: patients were 33 to 74 years old, median age: 57 years) were collected and processed at the Department of Obstetrics and Gynecology of the Medical University of Innsbruck, Austria between 1989 and 2010 as described recently [35]. Written informed consent was obtained from all patients before enrolment. The study was reviewed and approved by the Ethics committee of the Medical University of Innsbruck (reference number: AN2016-0024 358/4.13) and conducted in accordance with the Declaration of Helsinki. All samples were anonymized before the commencement of the analysis. All patients were monitored within the outpatient follow-up program of our department. The median observation period was 5.5 years (0.1 to 26.1). All patients were of Caucasian ethnicity. Clinicopathological features are shown in Table 1.

### RNA isolation and reverse transcription

Total cellular RNA extraction and reverse transcription were performed as previously described [35].

### Quantitative real time PCR

Primers and probes for the TATA box-binding protein (*TBP*; endogenous RNA-control) were used according to *Bieche et al.* [36]. Primers and probes for *PD-L1* (*CD274*) [GenBank: NM_014143.3] were determined with the assistance of the computer program Primer Express (Life Technologies, Carlsbad, CA, USA). BLASTN searches were conducted to confirm the total gene specificity of the nucleotide sequences chosen for the primers and probes. *PD-L1* forward primer: 5′-AATGATGGATGTGAAAAAATGTGG-3′; *PD-L1* reverse-primer: 5′-AATGCTGGATTACGTCTCCTCC-3′; *PD-L1* TaqMan probe: 5′-FAM-TCCAAGATACAAACTCAAAGAAGCAAAGTGATACACATT-3′-TAMRA. To prevent amplification of contaminating genomic DNA, the probe was placed at the junction between exons 6 and 7. Primers and probe for *IFNG* and *PD-1* were purchased from Applied Biosystems (Foster City, CA, USA, Applied Biosystems Assay ID: Hs00174143_m1 and Hs01550088_m1). PCR reactions were performed as previously described [35].

### Immunohistochemistry

Serial sections of the paraffin embedded material were cut at 2 µm and further processed using a BenchMark™ Ultra automated stainer (Roche Ventana). For the particular primary antibodies the following procedures were used: PD-L1 (clone 28-8; Abcam) diluted at 1:100 was incubated for 32 minutes and the OptiView™ DAB detection kit system CC1 was used for 36 minutes. PD-1 (clone NAT 105; Cell Marque AK) ready to use incubated for 32 minutes followed by the UltraView™ DAB detection kit CC2 for 44 minutes. For counterstaining hematoxylin was used. The evaluation of immunohistochemistry was performed semiquantitatively.

### Mutation analysis

Genomic DNA from 158 pulverized, quick-frozen OC specimens was isolated using the DNeasy tissue-kit (Qiagen, Hilden, Germany). Targeted NGS was performed using the TruSight Cancer sequencing panel (Illumina, San Diego, USA). The analyses were performed on the Illumina MiSequ^®^ and the NextSeq system (Illumina, CA, USA). After sequencing, mutations in *BRCA1, BRCA2* and *TP53* were identified with the help of NextGene and Geneticist Assistant softwares. Pathogenicity for new mutations that were not found in the database was determined and categorized using prediction tools like SIFT, alignGVGD, mutation taster.

### Statistical analysis

The non-parametric Mann-Whitney *U* test or Kruskal–Wallis test were applied to test for statistical significance between two groups or more than two groups, respectively. For parametric sample sets student’s two-tailed *t*-test was applied to test for statistical significance between two groups. The correlations between *PD-1*, *PD-L1* and *IFNγ* mRNA expression were assessed by Spearman rank correlation analyses (log-transformed data were analyzed by Pearson’s correlation analyses). Progression free survival (PFS) was defined as the time from diagnosis of the primary to tumor to the histopathological confirmation of recurrence or metastases and overall survival (OS) as the time from diagnosis of the primary to tumor to death from any cause or to the last clinical inspection. Univariate Kaplan-Meier analyses and multivariable Cox survival analyses were used to explore the association of *PD-1* and *PD-L1* expression with PFS and OS (the *p*-value cut-off for inclusion to the multivariable Cox analysis was 0.2). For survival analyses, patients were dichotomized into low and high mRNA expression level groups by the optimal cut-off expression value calculated by the Youden’s index [17]. *P*-values less than 0.05 were considered as statistically significant. Statistical analysis was performed using SPSS statistical software (version 20.0.0; SPSS Inc., Chicago, IL, USA).

## SUPPLEMENTARY MATERIALS FIGURES



## Abbreviations

BRCA: breast cancer gene
FIGO: Fédération Internationale de Gynécologie et d’Obstétrique
HGSOC: high grade serous ovarian cancer
IFN: Interferon
LGSOC: low grade serous ovarian cancer
NSCLC: non-small cell lung cancer
OC: ovarian cancer
OS: overall survival
PARP: poly-ADP-ribose polymerase
PD-1: programmed cell death 1
PD-L1: programmed cell death ligand-1
TILs: tumor infiltrating lymphocytes
TP53: tumor protein p53
PFS: progression free survival

## References

[1] Cannistra SA (2004). Cancer of the ovary. N Engl J Med.

[2] Burger RA, Brady MF, Bookman MA, Fleming GF, Monk BJ, Huang H, Mannel RS, Homesley HD, Fowler J, Greer BE, Boente M, Birrer MJ, Liang SX, Gynecologic Oncology Group (2011). Incorporation of bevacizumab in the primary treatment of ovarian cancer. N Engl J Med.

[3] Baldwin LA, Huang B, Miller RW, Tucker T, Goodrich ST, Podzielinski I, DeSimone CP, Ueland FR, van Nagell JR, Seamon LG (2012). Ten-year relative survival for epithelial ovarian cancer. Obstet Gynecol.

[4] Martin SD, Coukos G, Holt RA, Nelson BH (2015). Targeting the undruggable: immunotherapy meets personalized oncology in the genomic era. Ann Oncol.

[5] Hodi FS, O’Day SJ, McDermott DF, Weber RW, Sosman JA, Haanen JB, Gonzalez R, Robert C, Schadendorf D, Hassel JC, Akerley W, van den Eertwegh AJ, Lutzky J (2010). Improved survival with ipilimumab in patients with metastatic melanoma. N Engl J Med.

[6] Mittica G, Genta S, Aglietta M, Valabrega G (2016). Immune Checkpoint Inhibitors: A New Opportunity in the Treatment of Ovarian Cancer?. Int J Mol Sci.

[7] Iwai Y, Ishida M, Tanaka Y, Okazaki T, Honjo T, Minato N (2002). Involvement of PD-L1 on tumor cells in the escape from host immune system and tumor immunotherapy by PD-L1 blockade. Proc Natl Acad Sci U S A.

[8] Zou W, Chen L (2008). Inhibitory B7-family molecules in the tumour microenvironment. Nat Rev Immunol.

[9] Topalian SL, Hodi FS, Brahmer JR, Gettinger SN, Smith DC, McDermott DF, Powderly JD, Carvajal RD, Sosman JA, Atkins MB, Leming PD, Spigel DR, Antonia SJ (2012). Safety, activity, and immune correlates of anti-PD-1 antibody in cancer. N Engl J Med.

[10] Zhang L, Conejo-Garcia JR, Katsaros D, Gimotty PA, Massobrio M, Regnani G, Makrigiannakis A, Gray H, Schlienger K, Liebman MN, Rubin SC, Coukos G (2003). Intratumoral T cells, recurrence, and survival in epithelial ovarian cancer. N Engl J Med.

[11] Hamanishi J, Mandai M, Iwasaki M, Okazaki T, Tanaka Y, Yamaguchi K, Higuchi T, Yagi H, Takakura K, Minato N, Honjo T, Fujii S (2007). Programmed cell death 1 ligand 1 and tumor-infiltrating CD8+ T lymphocytes are prognostic factors of human ovarian cancer. Proc Natl Acad Sci U S A.

[12] Garcia-Diaz A, Shin DS, Moreno BH, Saco J, Escuin-Ordinas H, Rodriguez GA, Zaretsky JM, Sun L, Hugo W, Wang X, Parisi G, Saus CP, Torrejon DY (2017). Interferon Receptor Signaling Pathways Regulating PD-L1 and PD-L2 Expression. Cell Rep.

[13] Abiko K, Matsumura N, Hamanishi J, Horikawa N, Murakami R, Yamaguchi K, Yoshioka Y, Baba T, Konishi I, Mandai M (2015). IFN-gamma from lymphocytes induces PD-L1 expression and promotes progression of ovarian cancer. Br J Cancer.

[14] Rizvi NA, Hellmann MD, Snyder A, Kvistborg P, Makarov V, Havel JJ, Lee W, Yuan J, Wong P, Ho TS, Miller ML, Rekhtman N, Moreira AL (2015). Cancer immunology. Mutational landscape determines sensitivity to PD-1 blockade in non-small cell lung cancer. Science.

[15] Snyder A, Makarov V, Merghoub T, Yuan J, Zaretsky JM, Desrichard A, Walsh LA, Postow MA, Wong P, Ho TS, Hollmann TJ, Bruggeman C, Kannan K (2014). Genetic basis for clinical response to CTLA-4 blockade in melanoma. N Engl J Med.

[16] Le DT, Uram JN, Wang H, Bartlett BR, Kemberling H, Eyring AD, Skora AD, Luber BS, Azad NS, Laheru D, Biedrzycki B, Donehower RC, Zaheer A (2015). PD-1 Blockade in Tumors with Mismatch-Repair Deficiency. N Engl J Med.

[17] Youden WJ (1950). Index for rating diagnostic tests. Cancer.

[18] Walsh T, Casadei S, Lee MK, Pennil CC, Nord AS, Thornton AM, Roeb W, Agnew KJ, Stray SM, Wickramanayake A, Norquist B, Pennington KP, Garcia RL (2011). Mutations in 12 genes for inherited ovarian, fallopian tube, and peritoneal carcinoma identified by massively parallel sequencing. Proc Natl Acad Sci U S A.

[19] Strickland KC, Howitt BE, Shukla SA, Rodig S, Ritterhouse LL, Liu JF, Garber JE, Chowdhury D, Wu CJ, D’Andrea AD, Matulonis UA, Konstantinopoulos PA (2016). Association and prognostic significance of BRCA1/2-mutation status with neoantigen load, number of tumor-infiltrating lymphocytes and expression of PD-1/PD-L1 in high grade serous ovarian cancer. Oncotarget.

[20] Koshiyama M, Matsumura N, Konishi I (2014). Recent concepts of ovarian carcinogenesis: type I and type II. Biomed Res Int.

[21] Dong ZY, Zhong WZ, Zhang XC, Su J, Xie Z, Liu SY, Tu HY, Chen HJ, Sun YL, Zhou Q, Yang JJ, Yang XN, Lin JX (2017). Potential Predictive Value ofTP53andKRASMutation Status for Response to PD-1 Blockade Immunotherapy in Lung Adenocarcinoma. Clin Cancer Res.

[22] Wang X, Teng F, Kong L, Yu J (2016). PD-L1 expression in human cancers and its association with clinical outcomes. Onco Targets Ther.

[23] Darb-Esfahani S, Kunze CA, Kulbe H, Sehouli J, Wienert S, Lindner J, Budczies J, Bockmayr M, Dietel M, Denkert C, Braicu I, Johrens K (2016). Prognostic impact of programmed cell death-1 (PD-1) and PD-ligand 1 (PD-L1) expression in cancer cells and tumor-infiltrating lymphocytes in ovarian high grade serous carcinoma. Oncotarget.

[24] Webb JR, Milne K, Kroeger DR, Nelson BH (2016). PD-L1 expression is associated with tumor-infiltrating T cells and favorable prognosis in high-grade serous ovarian cancer. Gynecol Oncol.

[25] Zheng H, Liu X, Zhang J, Rice SJ, Wagman M, Kong Y, Zhu L, Zhu J, Joshi M, Belani CP (2016). Expression of PD-1 on CD4+ T cells in peripheral blood associates with poor clinical outcome in non-small cell lung cancer. Oncotarget.

[26] Hsu MC, Hsiao JR, Chang KC, Wu YH, Su IJ, Jin YT, Chang Y (2010). Increase of programmed death-1-expressing intratumoral CD8 T cells predicts a poor prognosis for nasopharyngeal carcinoma. Mod Pathol.

[27] Thompson RH, Dong H, Lohse CM, Leibovich BC, Blute ML, Cheville JC, Kwon ED (2007). PD-1 is expressed by tumor-infiltrating immune cells and is associated with poor outcome for patients with renal cell carcinoma. Clin Cancer Res.

[28] Alberts DS, Marth C, Alvarez RD, Johnson G, Bidzinski M, Kardatzke DR, Bradford WZ, Loutit J, Kirn DH, Clouser MC, Markman M, GRACES Clinical Trial Consortium (2008). Randomized phase 3 trial of interferon gamma-1b plus standard carboplatin/paclitaxel versus carboplatin/paclitaxel alone for first-line treatment of advanced ovarian and primary peritoneal carcinomas: results from a prospectively designed analysis of progression-free survival. Gynecol Oncol.

[29] Jurado JO, Alvarez IB, Pasquinelli V, Martinez GJ, Quiroga MF, Abbate E, Musella RM, Chuluyan HE, Garcia VE (2008). Programmed death (PD)-1:PD-ligand 1/PD-ligand 2 pathway inhibits T cell effector functions during human tuberculosis. J Immunol.

[30] Loke P, Allison JP (2003). PD-L1 and PD-L2 are differentially regulated by Th1 and Th2 cells. Proc Natl Acad Sci U S A.

[31] Bruggemann C, Kirchberger MC, Goldinger SM, Weide B, Konrad A, Erdmann M, Schadendorf D, Croner RS, Krahenbuhl L, Kahler KC, Hafner C, Leisgang W, Kiesewetter F (2017). Predictive value of PD-L1 based on mRNA level in the treatment of stage IV melanoma with ipilimumab. J Cancer Res Clin Oncol.

[32] Webb JR, Milne K, Nelson BH (2015). PD-1 and CD103 Are Widely Coexpressed on Prognostically Favorable Intraepithelial CD8 T Cells in Human Ovarian Cancer. Cancer Immunol Res.

[33] Maine CJ, Aziz NH, Chatterjee J, Hayford C, Brewig N, Whilding L, George AJ, Ghaem-Maghami S (2014). Programmed death ligand-1 over-expression correlates with malignancy and contributes to immune regulation in ovarian cancer. Cancer Immunol Immunother.

[34] Lee JM, Cimino-Mathews A, Peer CJ, Zimmer A, Lipkowitz S, Annunziata CM, Cao L, Harrell MI, Swisher EM, Houston N, Botesteanu DA, Taube JM, Thompson E (2017). Safety and Clinical Activity of the Programmed Death-Ligand 1 Inhibitor Durvalumab in Combination With Poly (ADP-Ribose) Polymerase Inhibitor Olaparib or Vascular Endothelial Growth Factor Receptor 1-3 Inhibitor Cediranib in Women’s Cancers: A Dose-Escalation, Phase I Study. J Clin Oncol.

[35] Goebel G, Berger R, Strasak AM, Egle D, Muller-Holzner E, Schmidt S, Rainer J, Presul E, Parson W, Lang S, Jones A, Widschwendter M, Fiegl H (2012). Elevated mRNA expression of CHAC1 splicing variants is associated with poor outcome for breast and ovarian cancer patients. Br J Cancer.

[36] Bieche I, Franc B, Vidaud D, Vidaud M, Lidereau R (2001). Analyses of MYC, ERBB2, and CCND1 genes in benign and malignant thyroid follicular cell tumors by real-time polymerase chain reaction. Thyroid.

