# Supplementary material for: BRCA1/2 and TP53 mutation status associates with PD-1 and PD-L1 expression in ovarian cancer
Source: Oncotarget. 2018 Apr 3;9(25):17501–11. doi: 10.18632/oncotarget.24770 (PMC5915132; doi:10.18632/oncotarget.24770)
Supplement: Supplementary file 1 [file oncotarget-09-17501-s001.pdf]

BRCA1/2 and *TP53* mutation status associates with *PD-1* and *PD-L1* expression in ovarian cancer

SUPPLEMENTARY MATERIALS

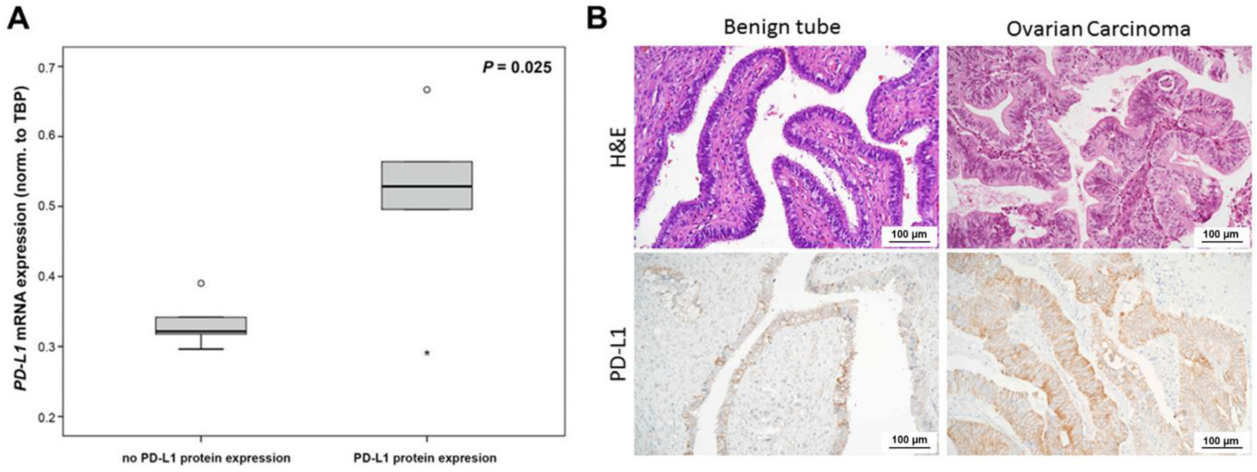

Supplementary Figure 1: *PD-L1* mRNA is associated with PD-L1 immunohistochemistry in non-malignant tissue. (**A**) *PD-L1* mRNA expression in patients with no immunohistochemical PD-L1 expression and cases with any immunohistochemical PD-L1 expression (n = 5). (**B**) Representative H&E section and PD-L1 immunohistochemistry of benign and malignant tissue.

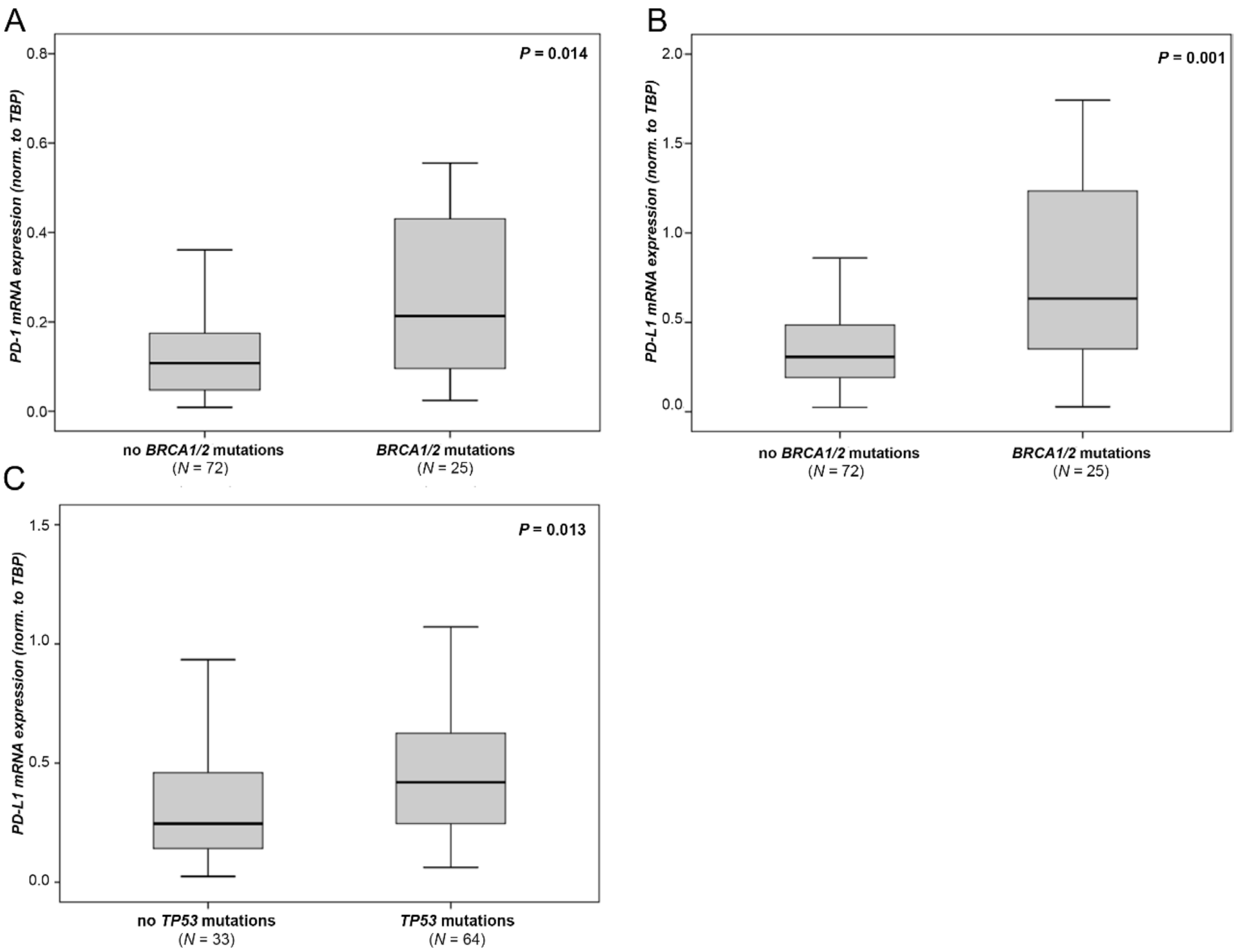

Supplementary Figure 2: *PD-1* and *PD-L1* mRNA expression according to genetic aberrations in HGSOC. *BRCA1/2* mutation data in association with (**A**) *PD-1* expression and (**B**) with *PD-L1* expression. (**C**) *TP53* mutation data in association with *PD-L1* expression. Expression values were normalized to *TBP* expression.
